# Correction: Genistein downregulates onco-miR-1260b and upregulates sFRP1 and Smad4 via demethylation and histone modification in prostate cancer cells

**DOI:** 10.1038/s41416-018-0146-2

**Published:** 2018-06-22

**Authors:** H Hirata, Y Hinoda, V Shahryari, G Deng, Y Tanaka, Z L Tabatabai, R Dahiya

**Affiliations:** 1 Department of Urology, San Francisco Veterans Affairs Medical Center and University of California at San Francisco, San Francisco, CA USA; 2 Department of Oncology and Laboratory Medicine, Yamaguchi University Graduate School of Medicine, Yamaguchi, Japan; 3 Department of Pathology, San Francisco Veterans Affairs Medical Center and University of California at San Francisco, San Francisco, CA USA

**Correction to:** *British Journal of Cancer* **110**, 1645–1654 (2014); 10.1038/bjc.2014.48; published online 6 February 2014

The authors report that there is a mistake in the representative picture of Fig. 4D (top row: PC3-miR1260b inh-0h) in the original version. The correct version of Fig. 4 with the original pictures for both PC3 miR-NC inh-0h and PC3-miR1260b inh-0h are provided below.

**Fig. 4:**
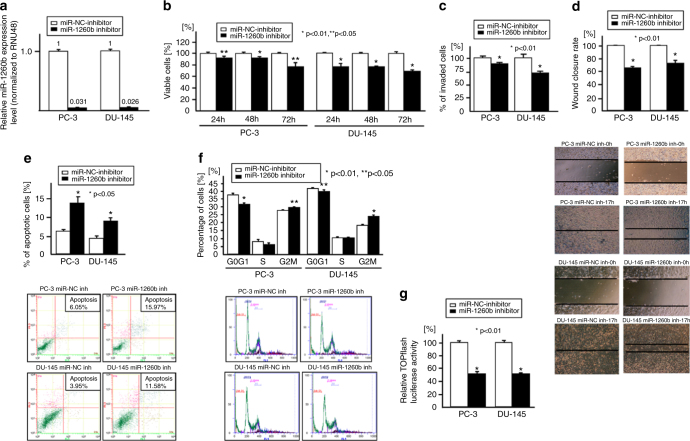
Effect of miR-1260b knockdown on prostate cancer cells (PC-3, DU-145). Two prostate cancer cell lines (PC-3 and DU-145) were transiently transfected with either miR-1260b inhibitor or miR-negative control (miR-NC inhibitor). **a** Relative miR-1260b expression (miR-NC inhibitor or miR-1260b inhibitor transfected PC cells), **b** cell viability assay (miR-NC inhibitor or miR-1260b inhibitor transfected PC cells), **c** invasion assay, **d** wound healing assay (16–17 h). **e** Apoptosis assay, f cell cycle analysis, **g** TCF reporter assay. Error bars represent ±s.d

